# 1′-Methyl-4′-(4-methyl­phen­yl)dispiro­[indane-2,3′-pyrrolidine-2′,3′′-indoline]-1,2′′-dione

**DOI:** 10.1107/S1600536812028012

**Published:** 2012-06-23

**Authors:** A. M. Moustafa, Adel S. Girgis, S. M. Shalaby, Edward R. T. Tiekink

**Affiliations:** a Solid State Department, Physics Division, National Research Centre, Dokki, Giza, Egypt; b Pesticide Chemistry Department, National Research Centre, Dokki, Giza, Egypt; c Department of Chemistry, University of Malaya, 50603 Kuala Lumpur, Malaysia

## Abstract

In the title mol­ecule, C_27_H_24_N_2_O_2_, the pyrrolidin-2-one ring is almost planar (r.m.s. deviation = 0.003 Å), the pyrrolidine ring has an envelope conformation (the N atom is the flap atom) and the cyclo­penta­none ring is twisted about the C_q_—C_m_ bond (q = quaternary and m = methylene). The ketone O atoms are directed to opposite sides of the mol­ecule. Supra­molecular chains along the *a* axis are formed in the crystal packing mediated by N—H⋯N and C—H⋯O inter­actions. These are connected into layers in the *ab* plane *via* C—H⋯π inter­actions.

## Related literature

For the biological activity of spiro­pyrrolidinyl-oxindolyl analogues, see: James & Williams (1972 ▶); Cui *et al.* (1996*a* ▶,*b* ▶); Palmisano *et al.* (1996 ▶); Garcia Prado *et al.* (2007 ▶); Girgis (2009*b* ▶); Girgis *et al.* (2012 ▶). For related structures, see: Moustafa *et al.* (2008 ▶); Li *et al.* (2008 ▶). For the synthesis, see: Girgis *et al.* (2009*a* ▶). For conformational analysis, see: Cremer & Pople (1975 ▶).
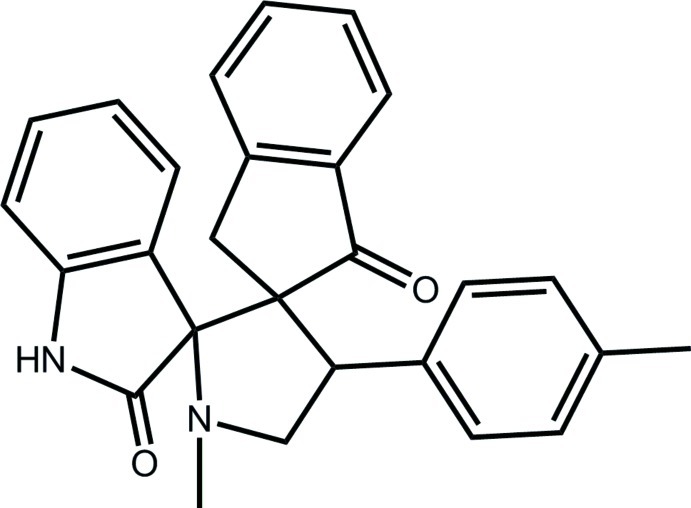


## Experimental

### 

#### Crystal data

C_27_H_24_N_2_O_2_
*M* *_r_* = 408.48
Triclinic, 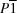

*a* = 6.2414 (2) Å
*b* = 11.3954 (5) Å
*c* = 15.5563 (7) Å
α = 78.386 (2)°
β = 87.165 (2)°
γ = 77.046 (2)°
*V* = 1056.17 (7) Å^3^
*Z* = 2
Mo *K*α radiation
μ = 0.08 mm^−1^
*T* = 293 K
0.25 × 0.08 × 0.05 mm

#### Data collection

Nonius KappaCCD diffractometer
Absorption correction: multi-scan (*SORTAV*; Blessing 1995 ▶) *T*_min_ = 0.852, *T*_max_ = 0.991
12225 measured reflections
4833 independent reflections
2335 reflections with *I* > 2σ(*I*)
*R*_int_ = 0.081

#### Refinement

*R*[*F* ^2^ > 2σ(*F* ^2^)] = 0.062
*wR*(*F* ^2^) = 0.154
*S* = 1.01
4833 reflections
285 parameters
1 restraint
H atoms treated by a mixture of independent and constrained refinement
Δρ_max_ = 0.21 e Å^−3^
Δρ_min_ = −0.23 e Å^−3^

### 

Data collection: *COLLECT* (Hooft, 1998 ▶); cell refinement: *SCALEPACK* (Otwinowski & Minor, 1997 ▶); data reduction: *DENZO* and *SCALEPACK*; program(s) used to solve structure: *SHELXS97* (Sheldrick, 2008 ▶); program(s) used to refine structure: *SHELXL97* (Sheldrick, 2008 ▶); molecular graphics: *ORTEP-3* (Farrugia, 1997 ▶) and *DIAMOND* (Brandenburg, 2006 ▶); software used to prepare material for publication: *publCIF* (Westrip, 2010 ▶).

## Supplementary Material

Crystal structure: contains datablock(s) global, I. DOI: 10.1107/S1600536812028012/qm2073sup1.cif

Structure factors: contains datablock(s) I. DOI: 10.1107/S1600536812028012/qm2073Isup2.hkl

Additional supplementary materials:  crystallographic information; 3D view; checkCIF report

## Figures and Tables

**Table 1:** Hydrogen-bond geometry (Å, °) *Cg*1 and *Cg*2 are the centroids of the C13–C18 and C21–C26 rings, respectively.

*D*—H⋯*A*	*D*—H	H⋯*A*	*D*⋯*A*	*D*—H⋯*A*
N1—H1*N*⋯N2^i^	0.86 (1)	2.28 (1)	3.098 (3)	160 (2)
C9—H9*B*⋯O1^ii^	0.97	2.45	3.241 (2)	138
C27—H27*B*⋯O2^i^	0.97	2.56	3.385 (2)	143
C24—H24⋯*Cg*1^iii^	0.93	2.78	3.620 (3)	150
C19—H19*B*⋯*Cg*2^iv^	0.96	2.97	3.761 (4)	140

Symmetry codes: (i) 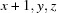; (ii) 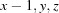; (iii) 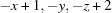; (iv) 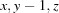.

**Fig. 1.**
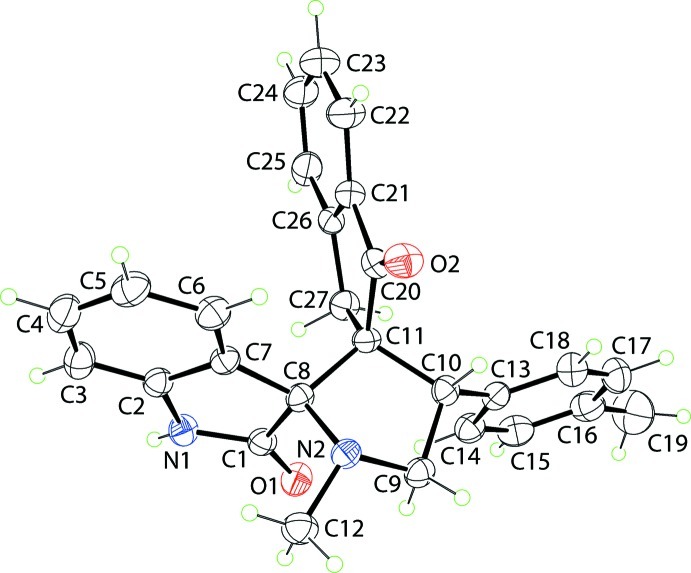
The molecular structure of (I) showing the atom-labelling scheme and displacement ellipsoids at the 35% probability level.

**Fig. 2.**
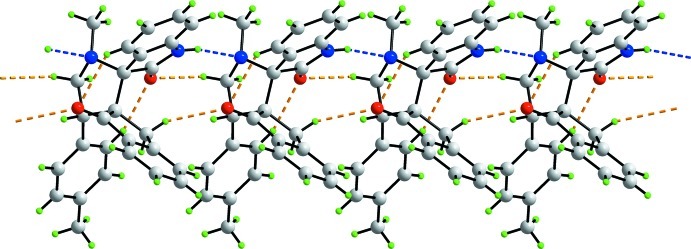
A view of the linear supramolecular chain propagated down the a axis via N—H···N hydrogen bonds (blue dashed lines) and C—H···O interactions (orange dashed lines) in the crystal structure of (I).

**Fig. 3.**
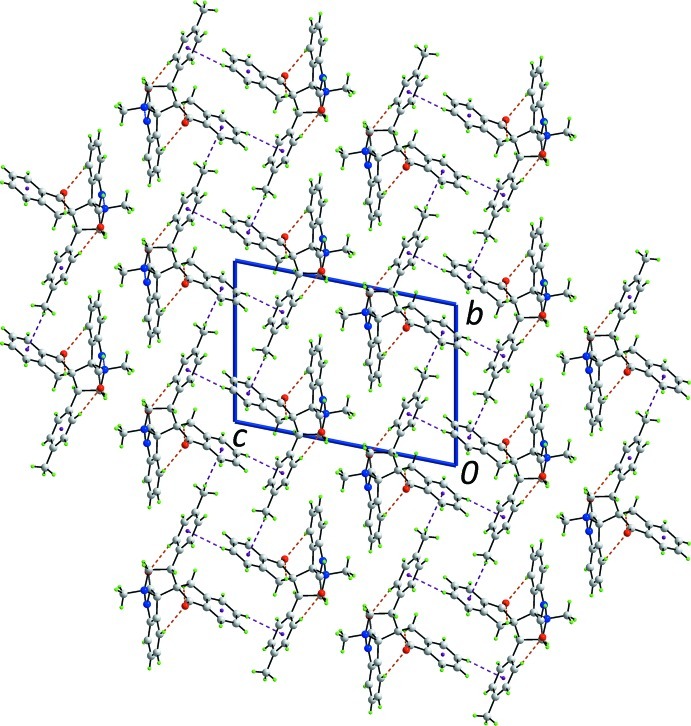
A view in projection down the a axis of the unit contents of (I). The N—H···N, C—H···O and C—H···π interactions are shown as blue, orange and purple dashed lines, respectively.

## supplementary crystallographic information

### Comment

Many spiropyrrolidinyl-oxindolyl analogues have been isolated from natural sources and identified as promising bio-active agents, *e.g*. spirotryprostatine A and spirotryprostatine B were found to be inhibitors of mammalian cell cycle at G2/*M* phase, from the secondary metabolites of *Aspergillus fugimatus* (Cui *et al.*, 1996*a*; Cui *et al.*, 1996*b*). Elacomine (James & Williams, 1972) was isolated from *Eleagnus commutata*, and horsfiline (Palmisano *et al.*, 1996), from *Horsfieldia superba*, a small Malaysian tree, extracts of which have found use in indigenous medicine. Mitraphylline was isolated from *Uncaria tomentosa* (cat's claw) and identified as an anti-tumour agent against human brain cancer cell lines, neuroblastoma SKN-BE(2) and malignant glioma GAMG (Garcia Prado *et al.*, 2007). One of the driving forces for initiating this work was our previous observations that compounds with alkaloid heterocyclic system skeletons, such as dispiro[1*H*-indene-2,3'-pyrrolidine-2',3''-[3*H*]indole]-1,2''(1''*H*)-diones and dispiro[3*H*-indole-3,2'-pyrrolidine-3',3''-piperidine]-2(1*H*),4''-diones, revealed promising anti-tumour properties against SK-MEL-2 (melanoma) cell line (Girgis, 2009*a*), and colon (HCT-116), breast (T-47D), leukemia [HL-60 (TB), MOLT-4, RPMI-8226] and prostate (PC-3) cell line cancers (Girgis, 2009*b*). Additionally, the analogue reported herein revealed mild anti-tumour properties against HCT116 (colon), HELA (cervical), HEPG2 (liver) and MCF7 (breast) human tumor cell lines (IC_50_ values = 33.81, 41.10, 23.89, 42.23 µ*M*, respectively), compared to that of the standard drug Doxorubicin (IC_50_ = 6.86, 7.71, 7.36, 5.46 µ*M*, respectively), utilizing the standard Sulfo-Rhodamine-B (SRB) method (Girgis *et al.*, 2012). With this background in mind, and in continuation of related structure studies (Moustafa *et al.*, 2008), herein we describe the crystal and molecular structure of the title compound, 2,3-dihydro-1'-methyl-4'-(4-methylphenyl)-dispiro-[1*H*-indene-2,3''-pyrrolidine-2',3''-[3*H*]indole]-1,2''(1''*H*)- dione, (I).

In (I), Fig. 1, the pyrrolidin-2-one ring is planar (r.m.s. deviation = 0.003 Å), the pyrrolidine ring has an envelope conformation where the N2 atom is the flap atom, and the cyclopentanone ring is twisted about the C11–C27 bond (Cremer & Pople, 1975). The ketone-O atoms are directed to opposite sides of the molecule. The overall conformation of the (I) matches that of the isoindole-1,3-dione derivative (Li *et al.*, 2008) with the greatest difference being found in the dihedral angle between the 2,3-dihydroisoindol-1-one and tolyl ring in (I), *i.e*. 23.97 (11)°, compared to 48.63 (7)° for the dihedral angle between the isoindole-1,3-dione and tolyl rings in the literature structure.

In the crystal packing, supramolecular chains along the *a* axis are formed by N—H···N hydrogen bonds complemented by C—H···O interactions with both carbonyl-O atoms participating in these contacts, Fig. 2 and Table 1. The chains are connected into supramolecular layers *via* C—H···π interactions, Table 1. Layers stack along the *c* axis without specific intermolecular interactions between them, Fig. 3.

### Experimental

The compound was prepared in accord with the literature procedure (Girgis *et al.*, 2009*a*). A mixture of 2(*E*)-2,3-dihydro-2-[(4-methylphenyl)methylene]-1*H*-inden-1-one 1 (1.17 g, 5 mmol), isatin 2 (0.81 g, 5.5 mmol) and sarcosine 3 (0.49 g, 5.5 mmol) in absolute ethanol (25 ml) was boiled under reflux. The separated solid was collected and re-crystallized from *n*-butanol by slow evaporation affording the title compound as colourless crystals, *M*.pt. 481–483 K. Yield: 80%.

### Refinement

Carbon-bound H-atoms were placed in calculated positions [C—H = 0.93 to 0.98 Å, *U*_iso_(H) = 1.2–1.5*U*_eq_(C)] and were included in the refinement in the riding model approximation. The N-bound H atom was refined with N—H = 0.86±0.01 Å, and with *U*_iso_(H) = 1.2*U*_eq_(N).

### Crystal data

**Table:**

C_27_H_24_N_2_O_2_	*Z* = 2
*M**_r_* = 408.48	*F*(000) = 432
Triclinic, *P*1	*D*_x_ = 1.284 Mg m^−^^3^
Hall symbol: -P 1	Mo *K*α radiation, λ = 0.71073 Å
*a* = 6.2414 (2) Å	Cell parameters from 12225 reflections
*b* = 11.3954 (5) Å	θ = 3.0–27.5°
*c* = 15.5563 (7) Å	µ = 0.08 mm^−^^1^
α = 78.386 (2)°	*T* = 293 K
β = 87.165 (2)°	Block, colourless
γ = 77.046 (2)°	0.25 × 0.08 × 0.05 mm
*V* = 1056.17 (7) Å^3^	

### Data collection

**Table:**

Nonius KappaCCD diffractometer	4833 independent reflections
Radiation source: fine-focus sealed tube	2335 reflections with *I* > 2σ(*I*)
Horizonally mounted graphite crystal monochromator	*R*_int_ = 0.081
Detector resolution: 9 pixels mm^-1^	θ_max_ = 27.5°, θ_min_ = 3.0°
φ & ω scans	*h* = −8→7
Absorption correction: multi-scan (*SORTAV*; Blessing 1995)	*k* = −11→14
*T*_min_ = 0.852, *T*_max_ = 0.991	*l* = −15→20
12225 measured reflections	

### Refinement

**Table:**

Refinement on *F*^2^	Primary atom site location: structure-invariant direct methods
Least-squares matrix: full	Secondary atom site location: difference Fourier map
*R*[*F*^2^ > 2σ(*F*^2^)] = 0.062	Hydrogen site location: inferred from neighbouring sites
*wR*(*F*^2^) = 0.154	H atoms treated by a mixture of independent and constrained refinement
*S* = 1.01	*w* = 1/[σ^2^(*F*_o_^2^) + (0.0631*P*)^2^ + 0.0079*P*] where *P* = (*F*_o_^2^ + 2*F*_c_^2^)/3
4833 reflections	(Δ/σ)_max_ < 0.001
285 parameters	Δρ_max_ = 0.21 e Å^−^^3^
1 restraint	Δρ_min_ = −0.23 e Å^−^^3^

### Special details

**Table:**

Geometry. All s.u.'s (except the s.u. in the dihedral angle between two l.s. planes) are estimated using the full covariance matrix. The cell s.u.'s are taken into account individually in the estimation of s.u.'s in distances, angles and torsion angles; correlations between s.u.'s in cell parameters are only used when they are defined by crystal symmetry. An approximate (isotropic) treatment of cell s.u.'s is used for estimating s.u.'s involving l.s. planes.
Refinement. Refinement of *F*^2^ against ALL reflections. The weighted *R*-factor *wR* and goodness of fit *S* are based on *F*^2^, conventional *R*-factors *R* are based on *F*, with *F* set to zero for negative *F*^2^. The threshold expression of *F*^2^ > 2σ(*F*^2^) is used only for calculating *R*-factors(gt) *etc*. and is not relevant to the choice of reflections for refinement. *R*-factors based on *F*^2^ are statistically about twice as large as those based on *F*, and *R*- factors based on ALL data will be even larger.

### Fractional atomic coordinates and isotropic or equivalent isotropic displacement parameters (Å^2^)

**Table:**

	*x*	*y*	*z*	*U*_iso_*/*U*_eq_	
O1	0.6791 (2)	0.04076 (16)	0.60805 (11)	0.0457 (5)	
O2	−0.0672 (2)	0.20742 (16)	0.77463 (11)	0.0496 (5)	
N1	0.6771 (3)	0.24335 (18)	0.60219 (12)	0.0393 (5)	
H1N	0.8160 (17)	0.240 (2)	0.5962 (15)	0.047*	
N2	0.1836 (3)	0.16036 (17)	0.58965 (11)	0.0343 (5)	
C1	0.5853 (3)	0.1437 (2)	0.61737 (14)	0.0351 (6)	
C2	0.5274 (3)	0.3484 (2)	0.61858 (14)	0.0363 (6)	
C3	0.5576 (4)	0.4665 (2)	0.60611 (16)	0.0481 (7)	
H3	0.6932	0.4843	0.5882	0.058*	
C4	0.3809 (4)	0.5584 (2)	0.62086 (16)	0.0530 (7)	
H4	0.3977	0.6389	0.6130	0.064*	
C5	0.1796 (4)	0.5311 (2)	0.64715 (16)	0.0511 (7)	
H5	0.0621	0.5937	0.6565	0.061*	
C6	0.1508 (4)	0.4119 (2)	0.65968 (16)	0.0441 (6)	
H6	0.0150	0.3944	0.6774	0.053*	
C7	0.3261 (3)	0.3190 (2)	0.64565 (14)	0.0340 (6)	
C8	0.3426 (3)	0.1840 (2)	0.64811 (14)	0.0323 (6)	
C9	0.2032 (3)	0.0270 (2)	0.61042 (14)	0.0381 (6)	
H9A	0.3381	−0.0154	0.5865	0.046*	
H9B	0.0793	0.0047	0.5876	0.046*	
C10	0.2052 (3)	−0.0030 (2)	0.71083 (14)	0.0352 (6)	
H10	0.0507	0.0121	0.7294	0.042*	
C11	0.3042 (3)	0.1001 (2)	0.73840 (14)	0.0320 (5)	
C12	0.2139 (4)	0.2079 (2)	0.49617 (15)	0.0470 (7)	
H12A	0.2007	0.2951	0.4866	0.070*	
H12B	0.1037	0.1902	0.4629	0.070*	
H12C	0.3571	0.1696	0.4777	0.070*	
C13	0.3025 (3)	−0.1357 (2)	0.74969 (15)	0.0368 (6)	
C14	0.5212 (3)	−0.1917 (2)	0.73573 (16)	0.0425 (6)	
H14	0.6127	−0.1465	0.7016	0.051*	
C15	0.6024 (4)	−0.3142 (2)	0.77248 (17)	0.0515 (7)	
H15	0.7496	−0.3489	0.7639	0.062*	
C16	0.4717 (4)	−0.3865 (2)	0.82152 (17)	0.0529 (7)	
C17	0.2557 (4)	−0.3311 (2)	0.83404 (17)	0.0548 (7)	
H17	0.1634	−0.3771	0.8667	0.066*	
C18	0.1730 (4)	−0.2087 (2)	0.79924 (16)	0.0471 (7)	
H18	0.0264	−0.1743	0.8093	0.057*	
C19	0.5612 (5)	−0.5205 (3)	0.8587 (2)	0.0880 (11)	
H19A	0.6731	−0.5539	0.8203	0.132*	
H19B	0.4444	−0.5639	0.8640	0.132*	
H19C	0.6229	−0.5290	0.9155	0.132*	
C20	0.1271 (3)	0.1721 (2)	0.79323 (15)	0.0368 (6)	
C21	0.2297 (4)	0.1875 (2)	0.87198 (15)	0.0395 (6)	
C22	0.1345 (4)	0.2491 (3)	0.93741 (17)	0.0552 (7)	
H22	−0.0138	0.2875	0.9357	0.066*	
C23	0.2674 (5)	0.2515 (3)	1.00543 (18)	0.0648 (8)	
H23	0.2083	0.2927	1.0500	0.078*	
C24	0.4867 (5)	0.1933 (3)	1.00763 (18)	0.0597 (8)	
H24	0.5738	0.1965	1.0536	0.072*	
C25	0.5791 (4)	0.1306 (2)	0.94330 (16)	0.0495 (7)	
H25	0.7268	0.0909	0.9459	0.059*	
C26	0.4486 (3)	0.1274 (2)	0.87431 (14)	0.0359 (6)	
C27	0.5096 (3)	0.0624 (2)	0.79916 (14)	0.0395 (6)	
H27A	0.5459	−0.0258	0.8200	0.047*	
H27B	0.6354	0.0871	0.7677	0.047*	

### Atomic displacement parameters (Å^2^)

**Table:**

	*U*^11^	*U*^22^	*U*^33^	*U*^12^	*U*^13^	*U*^23^
O1	0.0351 (9)	0.0427 (11)	0.0610 (12)	−0.0061 (8)	0.0061 (7)	−0.0184 (9)
O2	0.0313 (9)	0.0638 (12)	0.0546 (11)	−0.0040 (8)	0.0022 (7)	−0.0210 (9)
N1	0.0286 (10)	0.0458 (14)	0.0456 (12)	−0.0113 (10)	0.0011 (9)	−0.0106 (10)
N2	0.0328 (10)	0.0362 (12)	0.0346 (12)	−0.0074 (8)	−0.0043 (8)	−0.0077 (9)
C1	0.0329 (12)	0.0411 (16)	0.0336 (14)	−0.0103 (12)	−0.0009 (10)	−0.0098 (12)
C2	0.0377 (13)	0.0376 (15)	0.0346 (14)	−0.0091 (11)	−0.0036 (10)	−0.0078 (11)
C3	0.0484 (15)	0.0461 (18)	0.0523 (17)	−0.0180 (13)	−0.0077 (12)	−0.0054 (13)
C4	0.0685 (18)	0.0400 (17)	0.0524 (18)	−0.0164 (14)	−0.0126 (13)	−0.0054 (14)
C5	0.0603 (17)	0.0402 (18)	0.0508 (17)	−0.0013 (13)	−0.0068 (13)	−0.0132 (14)
C6	0.0415 (14)	0.0424 (17)	0.0482 (16)	−0.0053 (12)	−0.0008 (11)	−0.0125 (13)
C7	0.0349 (12)	0.0340 (15)	0.0332 (14)	−0.0064 (10)	−0.0031 (10)	−0.0077 (11)
C8	0.0268 (11)	0.0358 (14)	0.0363 (14)	−0.0081 (9)	−0.0015 (9)	−0.0100 (11)
C9	0.0325 (12)	0.0441 (16)	0.0410 (15)	−0.0103 (10)	−0.0031 (10)	−0.0132 (12)
C10	0.0276 (11)	0.0386 (15)	0.0408 (15)	−0.0086 (10)	0.0007 (9)	−0.0095 (11)
C11	0.0303 (11)	0.0361 (14)	0.0309 (13)	−0.0065 (10)	0.0008 (9)	−0.0109 (11)
C12	0.0536 (15)	0.0507 (17)	0.0370 (15)	−0.0110 (12)	−0.0052 (11)	−0.0085 (12)
C13	0.0389 (13)	0.0361 (15)	0.0378 (14)	−0.0104 (11)	−0.0020 (10)	−0.0101 (12)
C14	0.0408 (14)	0.0408 (16)	0.0463 (16)	−0.0063 (11)	0.0001 (11)	−0.0123 (13)
C15	0.0477 (15)	0.0485 (18)	0.0543 (18)	0.0036 (13)	−0.0028 (12)	−0.0161 (14)
C16	0.0678 (18)	0.0447 (18)	0.0440 (16)	−0.0070 (14)	−0.0060 (13)	−0.0077 (14)
C17	0.0659 (18)	0.0479 (19)	0.0518 (18)	−0.0228 (14)	0.0019 (13)	−0.0011 (14)
C18	0.0455 (14)	0.0460 (17)	0.0498 (16)	−0.0120 (12)	−0.0008 (11)	−0.0067 (13)
C19	0.109 (3)	0.048 (2)	0.091 (3)	−0.0015 (18)	−0.0049 (19)	0.0051 (19)
C20	0.0344 (13)	0.0390 (15)	0.0372 (14)	−0.0102 (10)	0.0032 (10)	−0.0066 (11)
C21	0.0463 (14)	0.0419 (16)	0.0330 (14)	−0.0139 (11)	0.0025 (11)	−0.0097 (12)
C22	0.0619 (16)	0.059 (2)	0.0473 (17)	−0.0111 (14)	0.0055 (13)	−0.0205 (15)
C23	0.085 (2)	0.074 (2)	0.0436 (18)	−0.0241 (17)	0.0074 (15)	−0.0248 (16)
C24	0.084 (2)	0.066 (2)	0.0372 (17)	−0.0302 (17)	−0.0091 (14)	−0.0109 (15)
C25	0.0588 (16)	0.0476 (17)	0.0435 (17)	−0.0183 (13)	−0.0117 (13)	−0.0017 (14)
C26	0.0443 (14)	0.0365 (15)	0.0290 (13)	−0.0151 (11)	−0.0003 (10)	−0.0041 (11)
C27	0.0351 (12)	0.0436 (16)	0.0400 (15)	−0.0082 (10)	−0.0037 (10)	−0.0083 (12)

### Geometric parameters (Å, º)

**Table:**

O1—C1	1.222 (3)	C12—H12B	0.9600
O2—C20	1.219 (2)	C12—H12C	0.9600
N1—C1	1.357 (3)	C13—C18	1.386 (3)
N1—C2	1.403 (3)	C13—C14	1.397 (3)
N1—H1N	0.860 (9)	C14—C15	1.387 (3)
N2—C12	1.465 (3)	C14—H14	0.9300
N2—C9	1.467 (3)	C15—C16	1.383 (3)
N2—C8	1.481 (3)	C15—H15	0.9300
C1—C8	1.562 (3)	C16—C17	1.377 (3)
C2—C3	1.375 (3)	C16—C19	1.508 (4)
C2—C7	1.396 (3)	C17—C18	1.380 (3)
C3—C4	1.385 (3)	C17—H17	0.9300
C3—H3	0.9300	C18—H18	0.9300
C4—C5	1.384 (3)	C19—H19A	0.9600
C4—H4	0.9300	C19—H19B	0.9600
C5—C6	1.383 (3)	C19—H19C	0.9600
C5—H5	0.9300	C20—C21	1.468 (3)
C6—C7	1.384 (3)	C21—C26	1.383 (3)
C6—H6	0.9300	C21—C22	1.387 (3)
C7—C8	1.511 (3)	C22—C23	1.385 (4)
C8—C11	1.573 (3)	C22—H22	0.9300
C9—C10	1.530 (3)	C23—C24	1.380 (4)
C9—H9A	0.9700	C23—H23	0.9300
C9—H9B	0.9700	C24—C25	1.375 (4)
C10—C13	1.509 (3)	C24—H24	0.9300
C10—C11	1.582 (3)	C25—C26	1.390 (3)
C10—H10	0.9800	C25—H25	0.9300
C11—C20	1.551 (3)	C26—C27	1.496 (3)
C11—C27	1.559 (3)	C27—H27A	0.9700
C12—H12A	0.9600	C27—H27B	0.9700
			
C1—N1—C2	111.53 (18)	N2—C12—H12C	109.5
C1—N1—H1N	124.0 (16)	H12A—C12—H12C	109.5
C2—N1—H1N	122.8 (16)	H12B—C12—H12C	109.5
C12—N2—C9	113.17 (18)	C18—C13—C14	117.0 (2)
C12—N2—C8	114.51 (16)	C18—C13—C10	120.33 (19)
C9—N2—C8	105.06 (15)	C14—C13—C10	122.6 (2)
O1—C1—N1	125.0 (2)	C15—C14—C13	120.4 (2)
O1—C1—C8	126.6 (2)	C15—C14—H14	119.8
N1—C1—C8	108.4 (2)	C13—C14—H14	119.8
C3—C2—C7	122.1 (2)	C16—C15—C14	122.2 (2)
C3—C2—N1	128.0 (2)	C16—C15—H15	118.9
C7—C2—N1	109.8 (2)	C14—C15—H15	118.9
C2—C3—C4	118.3 (2)	C17—C16—C15	117.1 (2)
C2—C3—H3	120.9	C17—C16—C19	121.5 (3)
C4—C3—H3	120.9	C15—C16—C19	121.4 (3)
C5—C4—C3	120.4 (3)	C16—C17—C18	121.5 (2)
C5—C4—H4	119.8	C16—C17—H17	119.3
C3—C4—H4	119.8	C18—C17—H17	119.3
C6—C5—C4	120.9 (2)	C17—C18—C13	121.8 (2)
C6—C5—H5	119.6	C17—C18—H18	119.1
C4—C5—H5	119.6	C13—C18—H18	119.1
C5—C6—C7	119.4 (2)	C16—C19—H19A	109.5
C5—C6—H6	120.3	C16—C19—H19B	109.5
C7—C6—H6	120.3	H19A—C19—H19B	109.5
C6—C7—C2	118.9 (2)	C16—C19—H19C	109.5
C6—C7—C8	131.74 (19)	H19A—C19—H19C	109.5
C2—C7—C8	109.20 (19)	H19B—C19—H19C	109.5
N2—C8—C7	113.33 (16)	O2—C20—C21	125.7 (2)
N2—C8—C1	112.07 (18)	O2—C20—C11	125.1 (2)
C7—C8—C1	101.14 (16)	C21—C20—C11	109.17 (18)
N2—C8—C11	102.74 (16)	C26—C21—C22	122.0 (2)
C7—C8—C11	118.25 (18)	C26—C21—C20	109.3 (2)
C1—C8—C11	109.57 (16)	C22—C21—C20	128.7 (2)
N2—C9—C10	103.46 (18)	C23—C22—C21	117.8 (2)
N2—C9—H9A	111.1	C23—C22—H22	121.1
C10—C9—H9A	111.1	C21—C22—H22	121.1
N2—C9—H9B	111.1	C24—C23—C22	120.5 (3)
C10—C9—H9B	111.1	C24—C23—H23	119.7
H9A—C9—H9B	109.0	C22—C23—H23	119.7
C13—C10—C9	114.3 (2)	C25—C24—C23	121.3 (3)
C13—C10—C11	118.71 (17)	C25—C24—H24	119.3
C9—C10—C11	104.82 (17)	C23—C24—H24	119.3
C13—C10—H10	106.0	C24—C25—C26	119.0 (2)
C9—C10—H10	106.0	C24—C25—H25	120.5
C11—C10—H10	106.0	C26—C25—H25	120.5
C20—C11—C27	102.80 (17)	C21—C26—C25	119.3 (2)
C20—C11—C8	110.42 (17)	C21—C26—C27	112.04 (19)
C27—C11—C8	112.89 (16)	C25—C26—C27	128.6 (2)
C20—C11—C10	107.69 (16)	C26—C27—C11	106.05 (17)
C27—C11—C10	119.51 (18)	C26—C27—H27A	110.5
C8—C11—C10	103.42 (17)	C11—C27—H27A	110.5
N2—C12—H12A	109.5	C26—C27—H27B	110.5
N2—C12—H12B	109.5	C11—C27—H27B	110.5
H12A—C12—H12B	109.5	H27A—C27—H27B	108.7

### Hydrogen-bond geometry (Å, º)

Cg1 and Cg2 are the centroids of the C13–C18 and C21–C26 rings, respectively.

**Table:**

*D*—H···*A*	*D*—H	H···*A*	*D*···*A*	*D*—H···*A*
N1—H1*N*···N2^i^	0.86 (1)	2.28 (1)	3.098 (3)	160 (2)
C9—H9*B*···O1^ii^	0.97	2.45	3.241 (2)	138
C27—H27*B*···O2^i^	0.97	2.56	3.385 (2)	143
C24—H24···*Cg*1^iii^	0.93	2.78	3.620 (3)	150
C19—H19*B*···*Cg*2^iv^	0.96	2.97	3.761 (4)	140

Symmetry codes: (i) *x*+1, *y*, *z*; (ii) *x*−1, *y*, *z*; (iii) −*x*+1, −*y*, −*z*+2; (iv) *x*, *y*−1, *z*.
